# Supplementary material for: Primary Cutaneous Multifocal Indolent CD8+ T-Cell Lymphoma: A Novel Primary Cutaneous CD8+ T-Cell Lymphoma
Source: Biomedicines. 2023 Feb 20;11(2):634. doi: 10.3390/biomedicines11020634 (PMC9953132; doi:10.3390/biomedicines11020634)
Supplement: Supplementary file 1 [file biomedicines-11-00634-s001.zip › biomedicines-2180560-supplementary.pdf]

SUPPLEMENTARY MATERIAL

Table S1: Immunohistochemistry Summary

**Immunohistochemical stain**	**CD3 (brown)/Ki-67 (magenta)**	**CD4 (brown)/Ki-67 (magenta)**	**CD8 (brown)/Ki67 (magenta)**	**TIA-1**	**CD68 KP-1**	**CD68 PGM1**	**LYSOZYME**	**PERFORIN (GLEN)**	**GRANZYME B (CHUS)**
**Platform**	Dako Omnis	Dako Omnis	Dako Omnis	Dako Omnis	Dako Omnis	Dako Omnis	Dako Omnis	Roche BenchMark ULTRA	X
**Technique**	EnVision FLEX, High pH (Dako Omnis), HRP (Horseradish Peroxidase)	EnVision FLEX, High pH (Dako Omnis), HRP (Horseradish Peroxidase)	EnVision FLEX, High pH (Dako Omnis), HRP (Horseradish Peroxidase)	EnVision FLEX, High pH (Dako Omnis), HRP (Horseradish Peroxidase)	EnVision FLEX, High pH (Dako Omnis), HRP (Horseradish Peroxidase)	EnVision FLEX, High pH (Dako Omnis), HRP (Horseradish Peroxidase)	EnVision FLEX, High pH (Dako Omnis), HRP (Horseradish Peroxidase)	Optiview DAB	X
**Manufacturer**	Dako Agilent/Dako Agilent	Roche/Dako Agilent	Dako Agilent /Dako Agilent	Biocare	Dako Agilent	Dako Agilent	Dako Agilent	Roche	X
**Clone**	Polyclonal/MIB-1	SP35/MIB-1	(C8/144B)/MIB-1	TIA-1	KP1	PGM1	Polyclonal	MRQ-23	X

For FasL and CCR4 we used antibodies from Abcam and Novus Biologicals, respectively, on a Ventana platform according to the manufacturer's instructions.

Figure S1: Flow cytometric analysis of lymphocytes extracted from skin

Figure S1a: year 2019

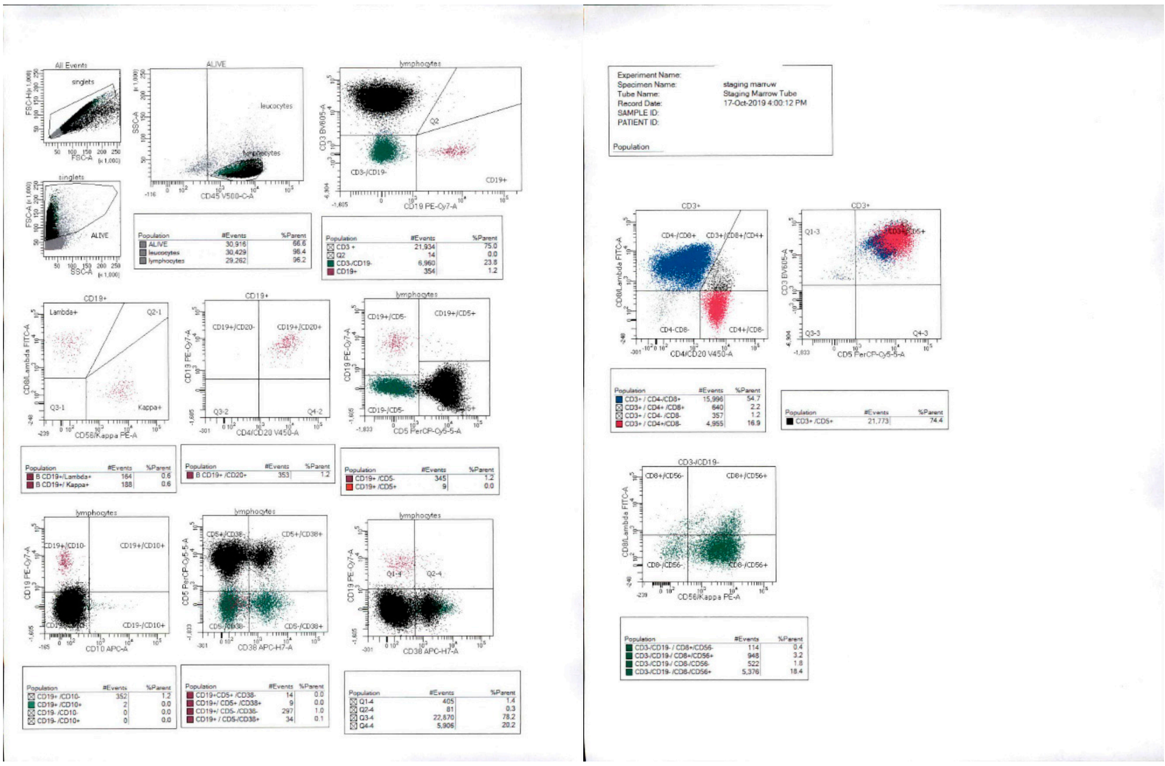

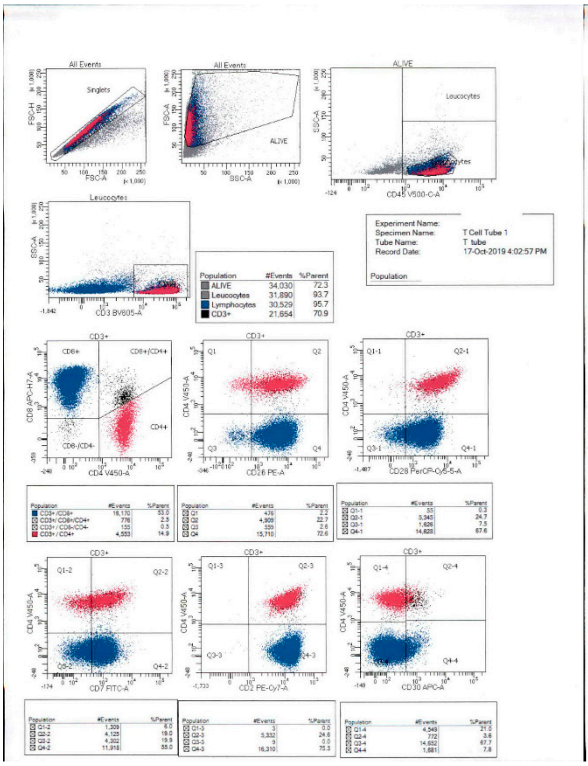
Experiment Name:
Specimen Name: T Cell Tube 1
Tube Name: T tube
Record Date: 17-Oct-2019 4:02:57 PM
Population
Population #Events %Parent
ALIVE 34,030 72.3
Leucocytes 31,890 93.7
Lymphocytes 30,529 95.7
CD3+ 21,654 70.9

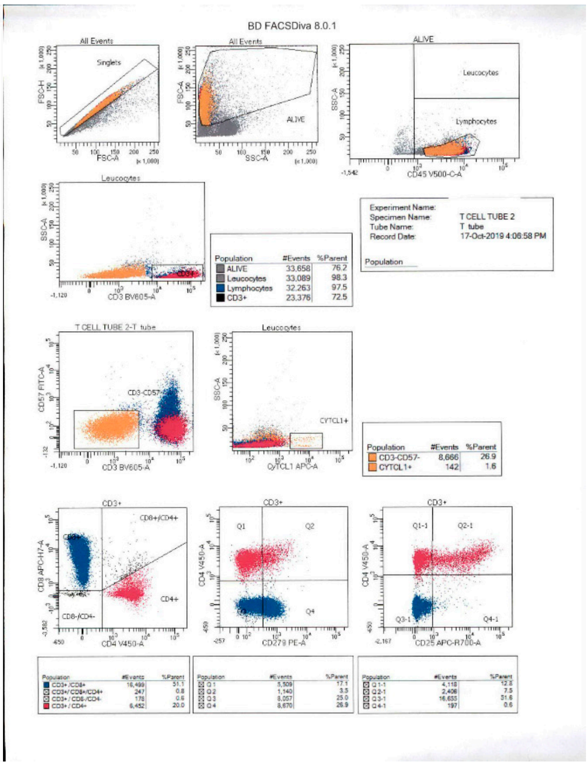
BD FACSDiva 8.0.1
Experiment Name:
Specimen Name: T CELL TUBE 2
Tube Name: T tube
Record Date: 17-Oct-2019 4:06:58 PM
Population
Population #Events %Parent
ALIVE 33,658 76.2
Leucocytes 33,089 98.3
Lymphocytes 32,263 97.5
CD3+ 23,376 72.5
T CELL TUBE 2-T tube
Population #Events %Parent
CD3-CD57- 8,666 26.9
CYTCL1+ 142 1.6

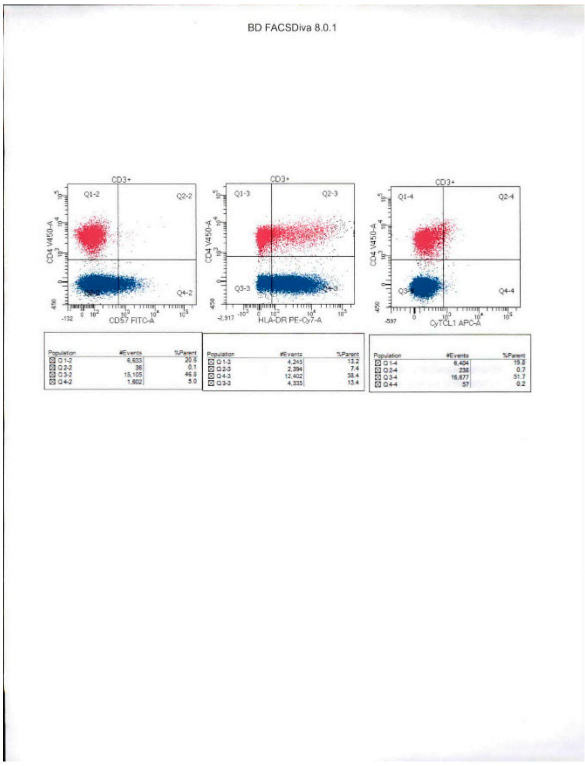
BD FACSDiva 8.0.1

Figure S1b: year 2021

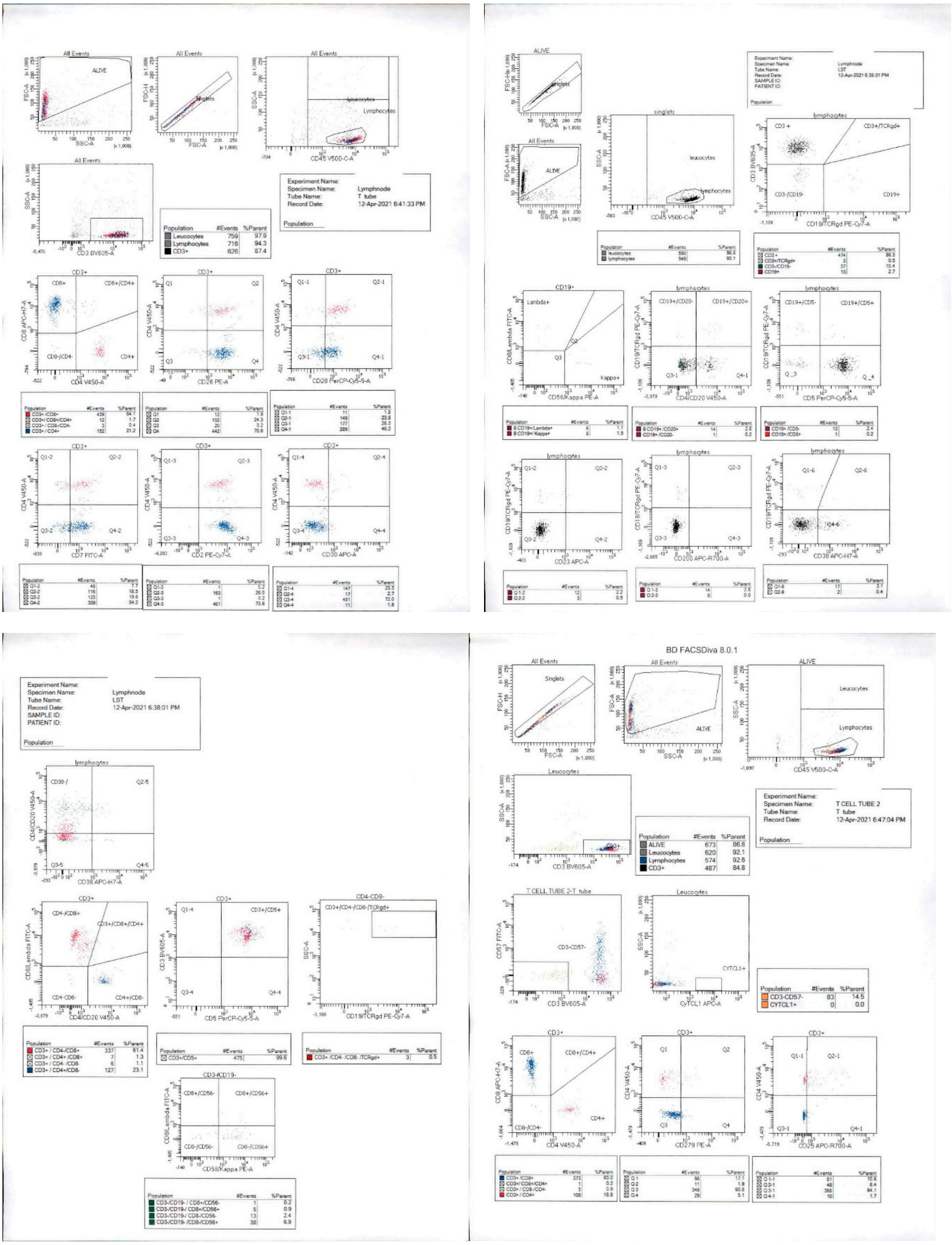

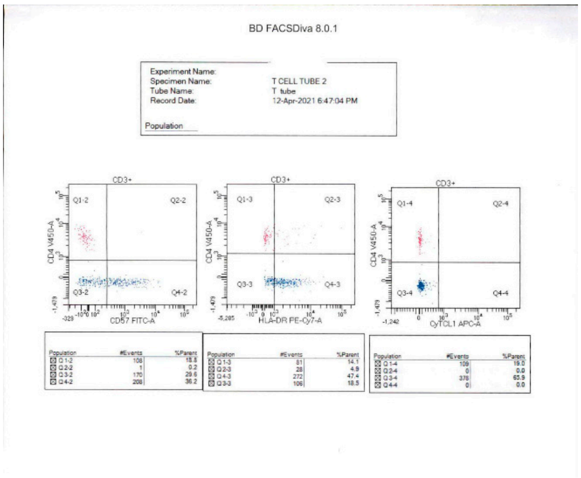

Figure S1 legend. a: 2019 FCM event (CD279/PD1a); b: 2021 FCM event (cf. Table 1).

Figure S2. Cell sorter gating and unstained control

Figure S2: FACS Gating Strategy

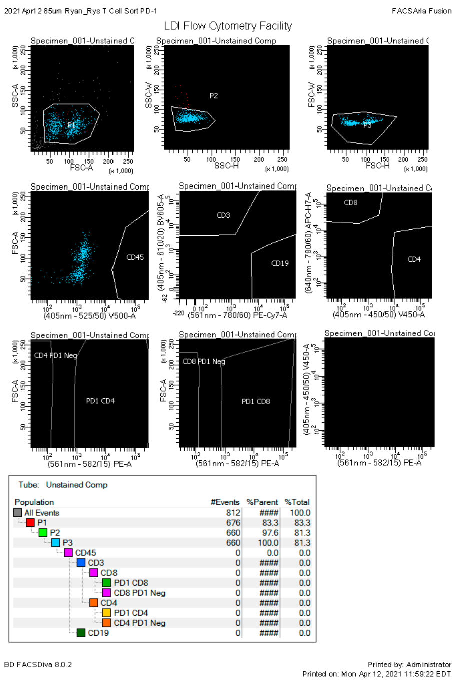

Figure S2: Cell sorter gating strategy with unstained control. a: Fluorescence-activated Cell Sorting (FACS)

Table S2: Detected Variants via Foundation Medicine One Heme Panel

Gene	Protein_Change	Variant Allele Frequency (VAF)	CDS Effect	Transcript	Position
CDKN2B	A64G	48.1	191C>G	NM_078487	CHR9:22008762
CUX1	R726Q	49.8	2177G>A	NM_001202543	CHR7:101844721
EPHB1	V333I	49.6	997G>A	NM_004441	CHR3:134851591
ERBB3	R521Q	52.5	1562G>A	NM_001982	CHR12:56487629
LRRK2	A2461V	45.7	7382C>T	NM_198578	CHR12:40758844
SETD2	L710S	47.5	2129T>C	NM_014159	CHR3:47163997
WHSC1	R944Q	47.9	2831G>A	NM_133335	CHR4:1957865

Table S2. Foundation Medicine One (FM1) Detected variants.

FM1 Panel gene list (Excel spreadsheet)

FoundationOne
Heme Supplemental-(
